# Designing functionality in perovskite thin films using ion implantation techniques: Assessment and insights from first-principles calculations

**DOI:** 10.1038/s41598-017-11158-4

**Published:** 2017-09-11

**Authors:** Vinit Sharma, Andreas Herklotz, Thomas Zac Ward, Fernando A. Reboredo

**Affiliations:** Materials Science and Technology Division, Oak Ridge National Laboratory, Oak Ridge, 37831-6056 TN USA

## Abstract

Recent experimental findings have demonstrated that low doses of low energy helium ions can be used to tailor the structural and electronic properties of single crystal films. These initial studies have shown that changes to lattice expansion were proposed to be the direct result of chemical pressure originating predominantly from the implanted He applying chemical pressure at interstitial sites. However, the influence of possible secondary knock-on damage arising from the He atoms transferring energy to the lattice through nuclear-nuclear collision with the crystal lattice remains largely unaddressed. Here, we study SrRuO_3_ to provide a comprehensive examination of the impact of common defects on structural and electronic properties. We found that, while interstitial He can modify the properties, a dose significantly larger than those reported in experimental studies would be required. Our study suggests that true origin of the observed changes is from combination of secondary defects created during He implantation. Of particular importance, we observe that different defect types can generate greatly varied local electronic structures and that the formation energies and migration energy barriers vary by defect type. Thus, we may have identified a new method of selectively inducing controlled defect complexes into single crystal materials.

## Introduction

The advent of epitaxial growth methods such as molecular beam epitaxy (MBE), metal organic chemical vapor deposition (MOCVD), pulse laser deposition (PLD)^1–6^ has allowed the fabrication of a large variety of artificial materials. In contrast with naturally occurring crystals, these growth techniques allow altering not only the chemistry but also the lattice parameters of custom designed artificial crystals. However, since growth temperatures in epitaxial methods are typically lower than for bulk materials, there is an underlying risk of introducing defects during growth. Therefore, while new materials often present novel properties, there is a constant debate in the literature to elucidate if these new properties are a result of the ideal material as designed or a consequence of persistent defects created during growth. There is currently no method of generating controlled defect concentrations in as-grown films which would allow direct control experiments.

In layer-by-layer epitaxial grown thin film geometries, the film is constrained along the in-plane lateral dimensions of the substrate. Meanwhile, the lattice parameter along the growth direction results from the unconstrained minimization of atomic forces. In recent experiments^5–8^, a surprising electronic and structural behavior of He-implanted perovskites have been reported. It was shown that by implanting noble He atoms into an epitaxial oxide film it is possible to expand the lattice in the growth direction and alter the symmetry of the crystal. The observed functional changes in these experiments were shown to correlate to structure driven changes to the base unit cell. However, even though these studies used relatively low acceleration energies on the implanted ions, these energies were still sufficiently high to induce knock-on damage in the underlying crystal. The question remains as to whether the exact mechanism driving lattice expansion was purely from interstitial He or whether secondary defects generated during the implantation process significantly contributed to lattice expansion. Simultaneously with a continuously controlled lattice expansion^7^, it was found that the band gap can be engineered^8^, with changes in electrical resistivity^6^, and structural distortion^5^. In highly correlated materials, a small change in lattice parameters can produce a significant change in electronic properties. However, in oxides very small concentration of defects, even undetectable using conventional techniques, can also significantly induce considerable changes in electric, dielectric, thermal and other properties^5–14^. However, the possible defects, including the effects of dose and defect formation, and the associated collateral effects involved in the He implantation process have not yet been thoroughly investigated.

Ion implantation has been widely used in the semiconductor industry for decades to selectively control electron/hole doping for device applications. Recently, experimental studies on ion implantation into more structurally and electronically complex materials have been undertaken^6–8, 15, 16^ in which defect generation has been used to control a variety of functional phenomena. Of particular interest, are recent findings demonstrating that low doses of low energy helium ions into single crystal films can be used to tailor the structural properties. These initial experimental studies have shown that crystal symmetry can be continuously controlled by applying increasingly large doses of He ions into a crystal. The observed changes in lattice structure were then observed to correlate with functional changes, such as metal-insulator transition temperature^7^ and optical bandgap^8^. In these preliminary experimental studies, the lattice changes were proposed to be the direct result of chemical pressure originating predominantly from the implanted He applying chemical pressure at interstitial sites. However, the influence of possible secondary knock-on damage arising from the He atoms transferring energy to the lattice through collision with the crystal lattice remains largely unaddressed.

Despite an intense experimental interest and potential applications, several of the critical questions pertaining to the observed electronic and structural properties in He implanted perovskite thin-films remain mostly unanswered. These questions include: (i) the identification of the stable interstitial sites of the implanted He atoms, (ii) the critical dose required for implanted He to change the electronic and structural properties of the system as observed experimentally, (iii) the possible mechanisms for He thermal migration and its activation energy barrier, (iv) which defects may appear as a collateral result of He implantation (at energies of ~4–10 keV) (v) the impact of defects on the structural and electronic properties of perovskite thin films (vi) the comparison between the annealing temperatures of intrinsic defects relative to the migration energy of He. Answering these questions will provide important fundamental insight into the mechanisms driving structural changes in the strain doped materials, while at the same time opening the door to understanding how specific defects might be controlled to gain a new level of defect designed engineering aimed at specific functionalities.

As a model system, we focus on SrRuO_3_ which was recently reported to undergo a continuously controlled shift from orthorhombic to tetragonal crystal phase under ion implantation^5^. In the initial experimental report, the quenching of octahedral rotation and increase in unit cell volume was suggested to be the direct result of interstitial He atoms applying chemical pressure to induce the observed structural changes. This explanation relies primarily on the impact of possible defects created by He implantation. In this work, we focus on a SrRuO_3_ model system to provide a comprehensive examination of the impact of common defects on structural and electronic properties, obtain calculated defect formation energies, and define defect migration barriers. We find that though He interstitial could produce the structural changes observed experimentally, the dose required is several orders of magnitude larger than the one reported experimentally. In contrast, several hundred secondary defects, with a formation energy below 5 eV, could be created by collisions with a single implanted He atom (at energies of ~4–10 keV). At low doses O, Sr, and, Ru interstitials produce much larger expansion in the lattice constants than He, while vacancies produce effects comparable to interstitial He. We find that the annealing temperatures required to heal intrinsic defects are much higher than the ones required for He to leave the sample. Our model also indicates that, while interstitial He can modify the crystal properties, a dose significantly larger than those reported in experimental studies would be required. The true origin of the observed structural changes is likely the result of a combination of secondary defects created during He implantation. Of particular importance, we observe that different defect types can generate greatly varied local electronic structures and that the formation energies and migration energy barriers vary by defect type. Thus, we may have identified a new method of selectively inducing controlled defect complexes into single crystal materials. Development of this approach would have a broad impact on both our ability to probe specific defect contributions in fundamental studies and allow a new level of control over functional properties driven by specific defect complexes.

The theoretical study was performed using density functional theory (DFT) calculations within generalized gradient approximation (GGA) functional^17^. (see Methods section) Here, in order to simulate the experiments^5, 8^, we have considered interstitial He and several defects in orthorhombic SrRuO_3_ films. To obtain an upper bound of the density of He, one can just take the dose in 5 × 10^15^ He/cm^2^ and assume an equal distribution across a 20–30 nm thick film where the unit-cell thickness is ~0.39 nm^5, 8^. This estimation ignores the He that either leaves the sample or could be trapped in the substrate. In experimental study, the upper bound for the He concentration in dosed film is found to be 0.15 He/unit-cell^5^. All structures considered are constrained on the (*a*-*b* plane) to match the parameters of SrTiO_3_ substrate. The *c* lattice parameter (perpendicular to the substrate) and the internal ionic coordinates are allowed to fully relax within the symmetry of the *Pbnm* space group. In past studies^18, 19^, this constrained-bulk approach has been successfully used for studying epitaxial strain effects in perovskite oxide thin films and superlattices.

## Results

The calculated formation energy is an estimate of the thermodynamic stability of a defect. Although the defect concentration is often determined by kinetics, there are important processes, eg. during prolonged anneals at high temperatures, where the concentration is governed primarily by defect formation energies. Thus the formation energies of individual native defects are quantities of central importance. Our computational exploration starts with the examination of relative stabilities of He interstitial atoms and their impact on the structural properties of the SrRuO_3_ films. Next, we determine the formation energies for different configurations of commonly considered intrinsic point defects, i.e., interstitials, substitutionals, and vacancies, which defects may appear as a collateral result of He implantation. Subsequently, we consider their effect on structural and electronic properties. Since, oxygen vacancies are the predominant defect in perovskite oxides^20–24^, in the present study, we also consider the interaction of oxygen vacancies (O_*vac*_) with He and other considered point defects.

### He substitutional and interstitial impurities

**Figure 1:**
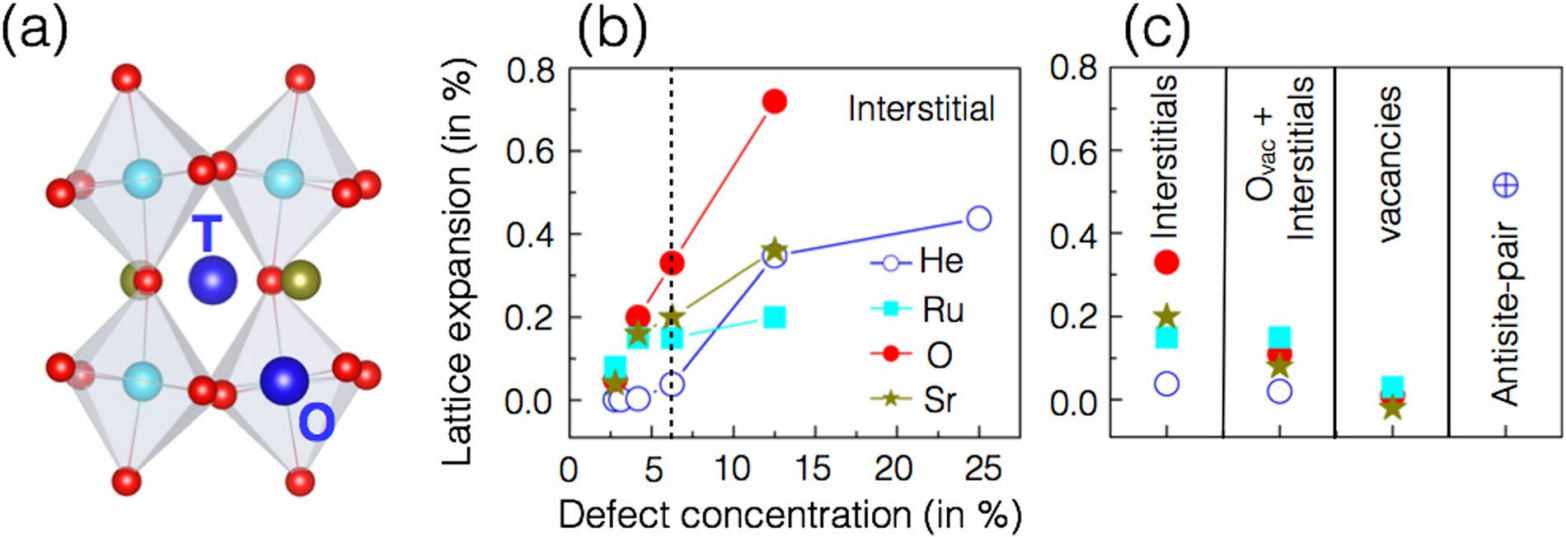
Effect of interstitial defects (including interstitials, self-interstitials, and vacancies) in orthorhombic SrRuO_3_. (**a**) Schematic representation of the orthorhombic SrRuO_3_, octahedral (O) and tetrahedral (T) interstitial sites are also labelled. He, O, Sr, and Ru atoms are shown in blue, red, dark yellow and cyan, respectively. (**b**) The uniaxial lattice expansion (along *c* axis) calculated in the SrRuO_3_ perovskite thin-films as a function of He and other intrinsic defect concentration. (**c**) Uniaxial lattice expansion resultant from various type of defects at the defect concentration of 6.25%.

Conventionally, He atoms may occupy either interstitial or substitutional sites. First of all, we compare various interstitial and substitutional defects formation energies, which are collected in Table 1. A corollary to the above finding is the recognition of the site preference of the He. From Table 1, it is evident that in the SrRuO_3_ thin-films He prefer to occupy the interstitial lattice sites. Next, we further compare the relative stabilities of various high symmetry interstitial sites. Orthorhombic SrRuO_3_ can accommodate interstitial at tetrahedral (1/4, 1/4, 1/4) and octahedral (0, 0, 1/2) sites (see Fig. 1(a)). Our calculated defect formation energies (also collected in Table 1) suggest that compared to tetrahedral interstice (2.56 eV) the octahedral interstice (2.23 eV) is energetically more favorable. As mentioned above, oxygen vacancies are the predominant defect in perovskite oxides^20–24^, thus we also explored He interstitial defects in the presence of adjunct O_*vac*_. Table 1 also shows that with an adjunct O_*vac*_ the preferred location of He interstitial is at the tetrahedral site.

**Table 1:** The calculated formation energies (in eV) of He impurities at substitutional and interestitial sites.

	Substitutional			Interstitial			
	O	Ru	Sr	Octahedral site		Tetrahedral site	
He	5.65	7.93	3.38	Interstitial	Interstitial + O_*vac*_	Interstitial	Interstitial + O_*vac*_
				2.23	2.17	2.56	1.97

The formation energies are determined as the difference of energy between the system with He and without He subtracting the energy of the He in vacuum. (see Supporting Information).

### Impact of the He implantation on the structural properties

Now, we turn to the question of the critical dose required for implanted He to change the structural properties. Based on ion implantation measurements, it was shown that small dose of He (<1%/volume or 4 × 10^20^ He/cm^3^) in SrRuO_3_ can introduce a structural distortion resulting in uniaxial lattice expansion along the *c* axis^5, 6^. Our calculations reveal that chemical pressure from interstitial He alone cannot give such a large lattice expansion. We find that even with more than double the He concentration reported in the experiment the effect on both lattice expansion and orthorhombic distortion are undetectable. Prior to investigating the collateral effects, we study the out-of-plane lattice expansion as a function of He impurity concentration is shown in Fig. 1(b). In order to model the experimental conditions, the calculations are performed with lattice vectors constrained to match a hypothetical substrate SrTiO_3_ and considered structures constrained on the perpendicular (*a*-*b* plane) to match the thin-film growth direction. The He atoms are introduced at the energetically favored octahedral interstitial sites. The *c* lattice parameter (perpendicular to the substrate) and the internal ionic coordinates are allowed to fully relax within the symmetry of the *Pbnm* space group. From Fig. 1(b), one can determine that the low He concentration reveals no lattice expansion along the thin-film growth direction. However, with increasing He impurity concentration (larger than >4%) a non-linear increase in out-of-plane lattice parameter is observed (Fig. 1(b)). In addition, Fig. 1(b) shows that to expand the lattice or tune the structural properties as observed experimentally a significantly larger He impurity concentration should be required.

### He migration barrier

**Figure 2:**
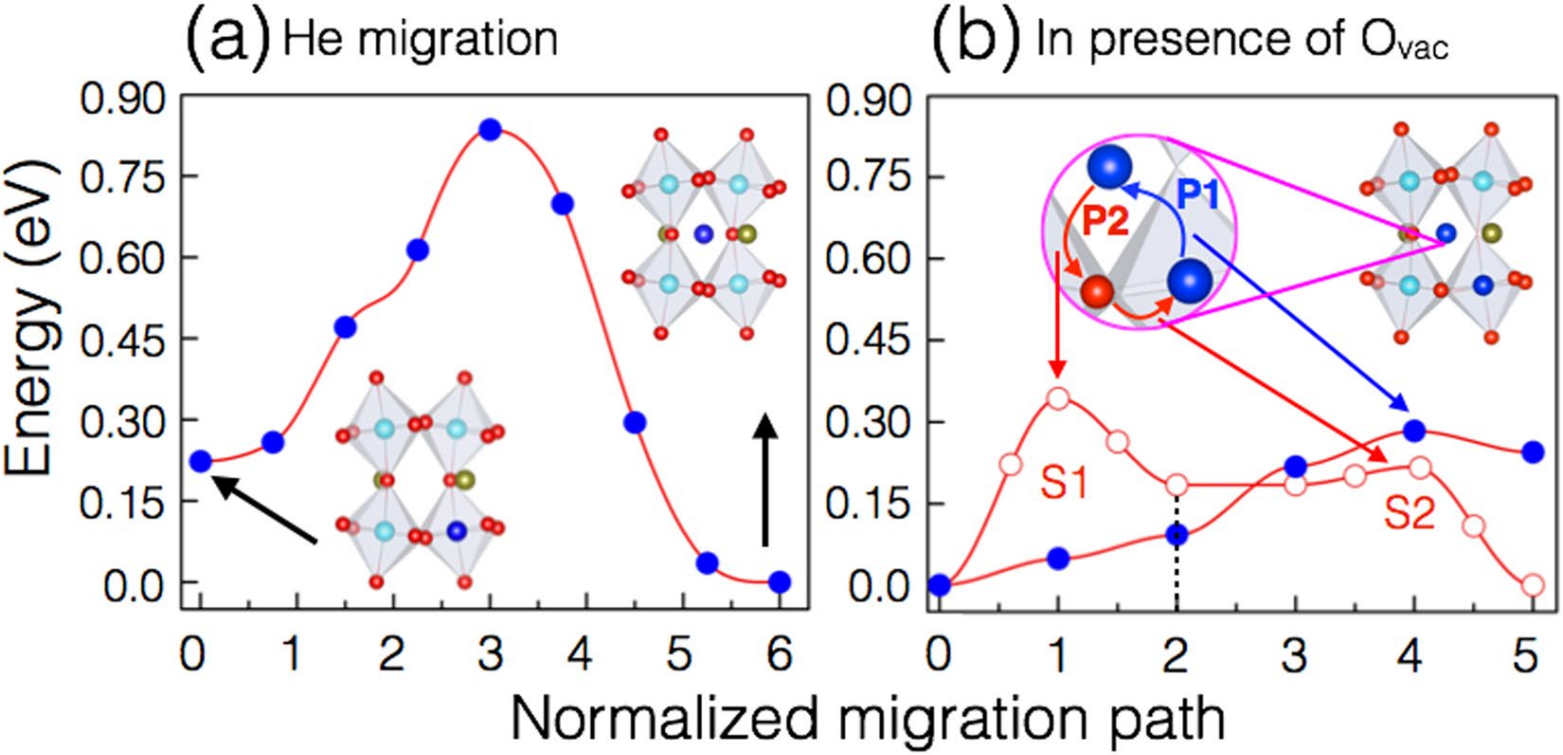
Computed He migration barriers. (**a**) He interstitial migration (filled blue circle) from tetrahedral site to octahedral site. A schematic illustration of the initial and final configurations used in the calculations is shown in the inset. (**b**) He migration in presence of O_*vac*_. The possible mechanisms for He migration are shown using blue (interstitial migration) and red (vacancy migration) arrows. P1 and P2 represent the possible minimum energy paths, considered for interstitial migration (filled blue circle) and vacancy migration (open red circle), respectively. In the case of vacancy migration, S1 corresponds to the first step where He atoms migrate to the neighboring O_*vac*_ site and in the next step (S2) He atoms migrate from O_*vac*_ site to the tetrahedral interstitial site. (**c**) O_*vac*_ migration to the energetically favorable octahedral interstitial site, with (open circle) and without (filled circles) oxygen vacancy. In the inset, schematic illustration of the initial and final configurations used in the barrier calculations is shown. The migration barriers are calculated using the generalized solid-state nudged elastic band method^25^. The lines are guides for the eye only.

Having established that He prefers to be on interstitial sites, we calculate the migration barriers to assess the mobility of He interstitials. At infinite dilution, He may jump to a neighboring interstitial. In present work, the migration barriers are calculated using the generalized solid-state nudged elastic band method^25^. The calculated hopping barrier for He interstitial is found to be ~0.83 eV (Fig. 2a). As mentioned above, oxygen vacancies are the predominant defect in perovskite oxides^20–24^. Next, to examine whether He migration is facilitated or suppressed by O_*vac*_, we explored the pathways for He migration in presence of O_*vac*_. Depending on the local He configuration, in presence of O_*vac*_ two mechanisms namely interstitial- and vacancy- migration paths are possible. In the case of the interstitial migration path, He atoms migrate through neighboring interstitial tetrahedral sites. In the presence of O_*vac*_, migration is a two-step process. A systematic capturing of both the migration paths (S1 and S2) is shown in Fig. 2b. In the first step (S1), He atoms migrate to the neighboring O_*vac*_ site. In the second step (S2), He atoms migrate from O_*vac*_ site to the tetrahedral interstitial site. With increasing O_*vac*_ concentration, the probability of multiple vacancies in the neighborhood of He sites increases. In this scenario, it is likely possible for He ion to pass through a neighboring O_*vac*_ site while jumping to a neighboring tetrahedral interstitial site.

Our calculations indicate that in the presence of oxygen vacancies (O_*vac*_) the barrier for the He interstitial migration is 0.28 eV, while the barrier in vacancy mechanism is 0.33 eV (for S1 step) and 0.21 eV (for S2 step). To compare with the experimental studies^5^, the small activation energies for He migration by interstitial and vacancy mechanisms are estimated for the limiting cases (i.e. low He and vacancy concentrations). Low migration energies (0.33 to 0.21 eV) also suggest that at low temperature, He migration is facilitated by oxygen vacancies (O_*vac*_).

### Intrinsic point defects

Now we consider an alternative possibility: that He implantation may generate by collisions intrinsic defect involving vacancies (O, Ru and Sr), interstitials (O, Ru and Sr) and/or anti-site defects. Our calculations indicate that the octahedral site is energetically favored for O, Ru and Sr interstitials. For each case, first, we calculated the defect formation energies under the two extreme conditions: the Sr-rich (SrO) limit and the Ru-rich (RuO_2_) limit. The formation energies (defined in Supporting Information) depend on the particular choice of the atomic chemical potentials^21–24^. In the present study, the chemical potential of the Ru and Sr atoms, (*μ*_*Ru*/*Sr*_), is defined using the total energies of the most stable cation oxides namely RuO_2_ or SrO for oxygen-rich conditions. The calculated formation energies are summarized in Table 2. Table 2 indicates that cation vacancies might be a dominant defect at equilibrium as their formation energies are lower than that of all other considered defects. However, during implantation vacancies and interstitials are expected to be formed in pairs.

**Table 2:** The calculated formation energies (in eV) for point defects, i.e., interstitial (octahedral and tetrahedral sites) and vacancies.

	Vacancies		Interstitial							
			Without Ovac				With Ovac			
	SrO- Rich	RuO_2_- Rich	SrO- Rich		RuO_2_- Rich		SrO- Rich		RuO_2_- Rich	
			Octa	Tetra	Octa	Tetra	Octa	Tetra	Octa	Tetra
Ru	2.90	2.32	12.03	12.45	11.45	11.87	14.93	13.58	13.77	13.00
Sr	1.44	2.02	13.57	12.22	14.15	12.80	15.01	15.88	15.17	16.46
O	4.38 (4.76^29^)		2.14 (Octa)				2.85 (Tetra)			
Antisite pair (Sr-Ru)	5.56									

The combined formation energy of a defect *and* an O_*vac*_, $({{\rm{E}}}_{f}^{d-{O}_{vac}})$, is determined as ${{\rm{E}}}_{f}^{d-{O}_{vac}}$ = ${{\rm{E}}}_{SRO}^{d-{O}_{vac}}$ − E_*SRO*_ − $({\mu }^{d}-\tfrac{1}{2}\,{\mu }_{{O}_{2}})$. The chemical potential of the Ru and Sr atoms, *μ* ^*d*^, is defined using the total energies of their respective most stable binary oxides namely RuO_2_ or SrO for oxygen rich conditions (also see Supporting Information). For comparison available experimental values are also given in parentheses.

Next, we examine the possibilities of interstitials (O, Ru, and Sr) and/or anti-site defects. As shown in Table 2, in pristine films, the oxygen interstitial is easier to form as compared to cation (Ru/Sr) interstitials. While studying these interstitial defects with adjunct oxygen vacancy, we found that the Ru interstitial is the energetically most favored interstitial defect. It is also evident from Table 2 that O_*vac*_ strongly binds and stabilizes the Sr and Ru interstitials.

Computational^26, 27^ and experimental^28, 29^ studies have shown that the presence of antisite defects significantly affects the electronic properties of perfect perovskites. In the case of SrRuO_3_, we found that the energy associated with the antisite pair (Sr-Ru) formation is higher than both cation and O_*vac*_ formation as well as the interstitial defects with adjacent O_*vac*_. However, at the same defect concentration, the antisite pair (Sr-Ru) produces the largest lattice expansion among all the defects studied in the present work. A comparison of the lattice expansion resultant from various defects (namely interstitial, interstitial with adjacent O_*vac*_, cation and O_*vac*_ and antisite defects) at 6.25% defect concentration is presented in Fig. 1(c).

### Effect of intrinsic point defects on structure properties

In order to understand the role of defects on structural properties, we calculated the lattice expansion as a function of interstitial (O, Ru and Sr) defect concentration. Figure 1(b) shows that with increasing defect concentration of O, Ru, and Sr interstitials, the lattice expands in the *c* direction. Our calculations indicate that a low concentration of interstitial defects (O, Ru and Sr) leads to larger lattice expansions than the He interstitial. At low defect concentrations, the recognizable expansion of the lattice is mainly the result of the intricate local chemical environment and distortion in the lattice introduced by the interstitial defects. Furthermore, due to the small ionic size of O atom and its low defect formation energy O interstitial defects are easier to form than Ru and Sr interstitials. (see Table 2) The oxygen interstitial breaks the octahedral connectivity, and, at higher concentrations, show an increase in the out-of-plane lattice expansion for oxygen interstitials (see Fig. 1(b)). Similar to the O interstitial, the Ru interstitial also breaks the octahedral connectivity. However, the strain resultant from the interstitial Ru impurity pushes the neighboring Ru atom to the next adjacent interstitial site and limits the out-of-plane lattice expansion. Our calculations suggest that the nature of the distortion introduced by the Sr interstitial to the system also contributes to the lattice expansion. The large ionic radius of Sr and high defect formation energies (see Table 2) suggest these defects are not easy to introduce at thermal equilibrium but may still be produced in collisions with He. It is also noteworthy that at higher concentrations, when a critical lattice expansion is reached, dislocations may form, however, this is outside the scope of the present work^30, 31^.

### Intrinsic Defect migration

**Figure 3:**
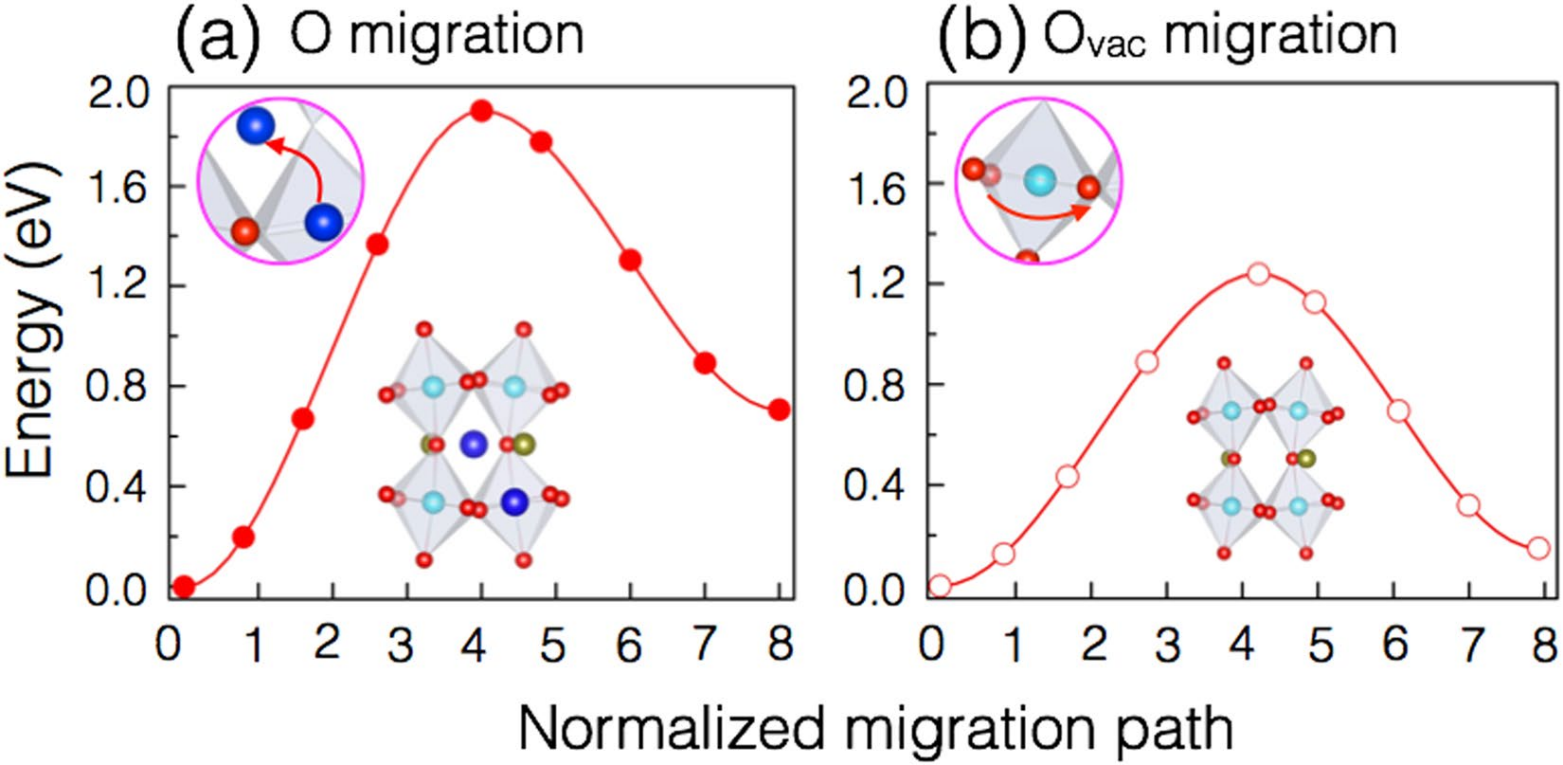
Computed migration barriers for oxygen interstitial migration and O_*vac*_ migration. (**a**) oxygen migration to octahedral interstitial site from tetrahedral interstitial site. (**b**) O_*vac*_ migration. In the inset, a schematic illustration of the initial and final configurations used in the calculations are shown. The migration barriers are calculated using the generalized solid-state nudged elastic band method^25^. The lines are guides for the eye only.

Ion-implantation experiments potentially can generate point defects^31, 32^. To understand the long-term behavior of He implantation damage in a SrRuO_3_ lattice, one requires an understanding of the migration behavior of all these defects. Therefore, first, we calculate the energy barriers for oxygen interstitial migration from the tetrahedral to the octahedral site and for the O_*vac*_ migration as shown in Fig. 3(a,b). The calculated migration barriers for oxygen interstitial (1.92 eV) and O_*vac*_ migration (1.23 eV) are found to be higher than the migration barrier of the He interstitial (0.83 eV). O_*vac*_ migration occurs more easily since the vacancies in the lattice facilitate the hopping between empty sites. Oxygen migration also depends on the disorder in the lattice. The presence of O_*vac*_ in the material may also enhance the diffusion of the defects like He by lowering their migration barriers, which is evident from both Figs 2(b) and 3(b).

**Figure 4:**
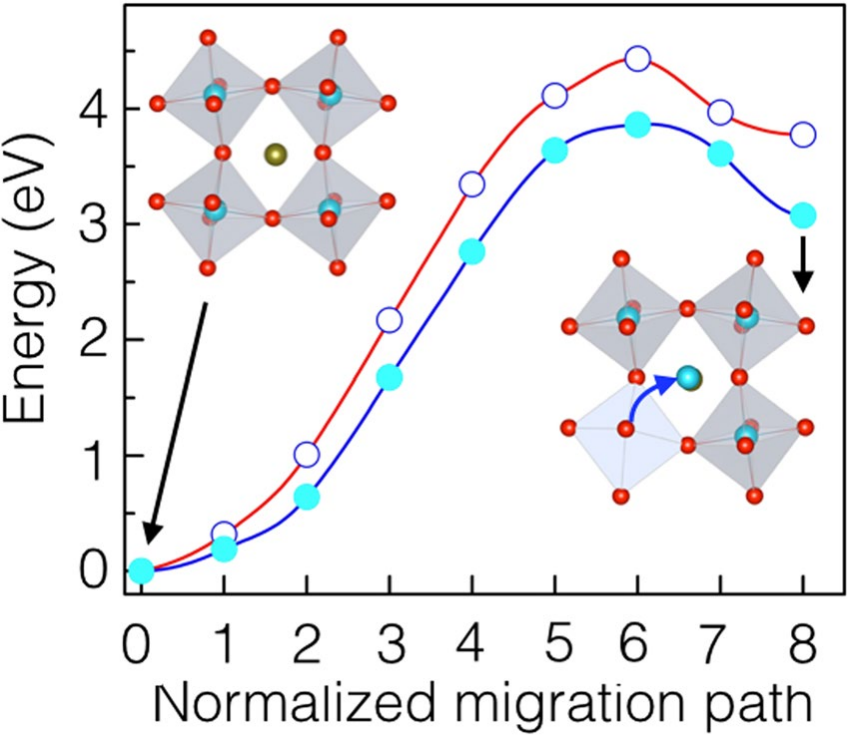
Ru interstitial migration. Ru migration to the energetically favorable octahedral interstitial site, with (open circle) and without (filled circles) oxygen vacancy. In the inset, a schematic illustration of the initial and final configurations used in the calculations is shown. The migration barriers are calculated using the generalized solid-state nudged elastic band method^25^. The lines are guides for the eye only.

Next, we calculate the energy barrier for Ru migration shown in Fig. 4. In pristine films, the calculated barrier for Ru migration is found to be 4.18 eV. It is interesting to note that in the presence of O_*vac*_, the calculated Ru migration barrier (3.6 eV) is also found to be lower than the pristine films.

The migration barriers for He (in presence of O_*vac*_) and O_*vac*_ migration are found to be lower than oxygen, O_*vac*_, and cation migration energies. Helium-O_*vac*_ clusters (with a formation energy 2.17 eV) likely form in the sample implanted with higher helium implantation energy, which is likely a combination of the initial defect structure and implantation depth^32, 33^. A deeper implantation allows helium to desorb from small vacancy sites and re-trap into helium-vacancy clusters that form at high temperature^32–34^. Our minimum energy path calculations suggest that the He atom migrates along the normal to the surface in a zigzag path. However, vacancies and interstitials constitute lattice strain normal to the surface and may influence the diffusivity of oxygen within the material^32–34^.

Owing to the lower activation energy, at low temperatures, the He interstitial migration is expected to be dominant for two reasons, (a) It is relatively faster than other defects. Due to the inert nature of He, the bonding of He interstitials to the surrounding atoms is weaker and (b) there are many more interstitial sites available for He migration than O or Ru vacancy sites. There may be other possible transition states, such as migration through Sr and Ru vacancy sites. Our calculations suggest that these have much higher energies (see Supporting Information). A corollary to the above finding is that upon annealing the migration of interstitial He atoms is predicted to be more rapid than the above-mentioned intrinsic defects. Higher migration barriers for oxygen and O_*vac*_ migration indicates that these defects may still remain in the material at higher temperatures when He is gone.

### Effect of defects on electronic structure of SrRuO_3_

**Figure 5:**
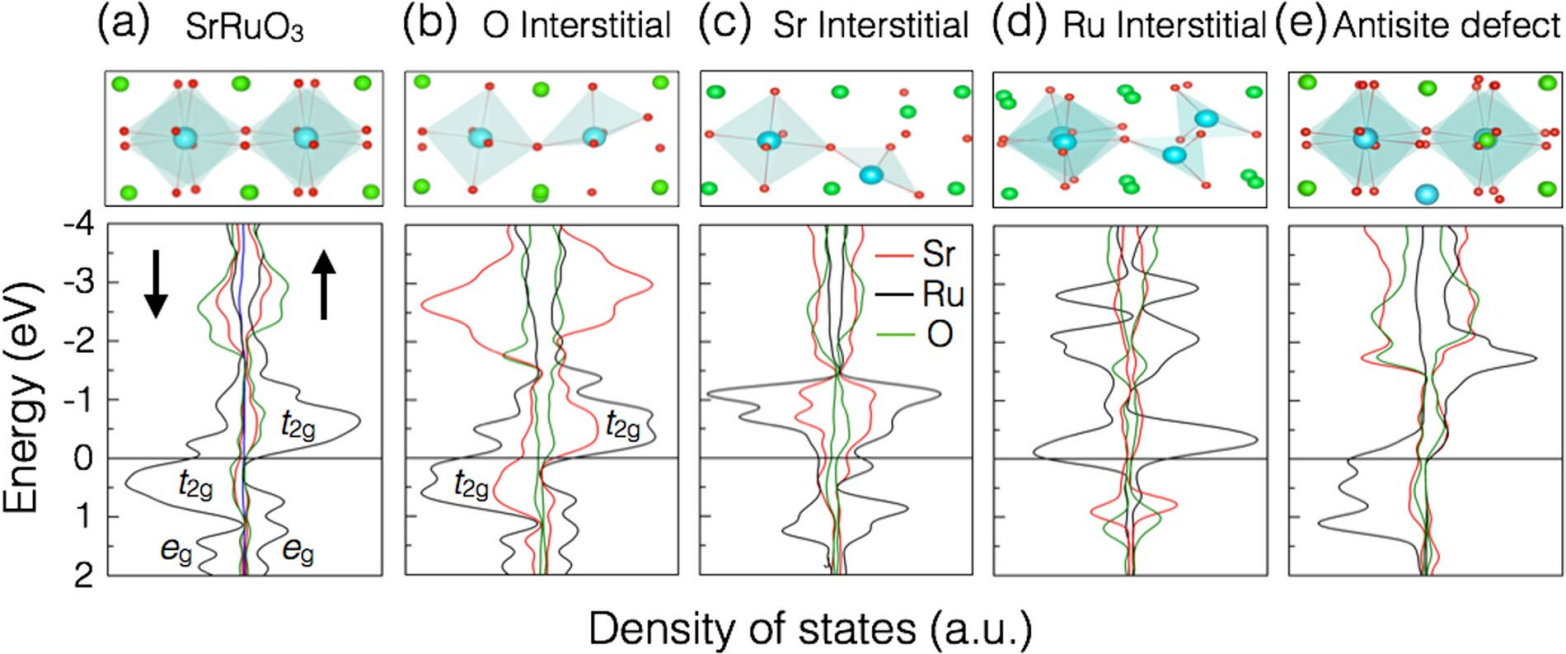
Effect of interstitial defects on local atomic environments in orthorhombic SrRuO_3_. Atom projected density of states for (**a**) pure SrRuO_3_ and interstitial defects (**b**) O interstitial (**c**) Ru interstitial (**d**) Sr interstitial and (**e**) anti site defects. Increased DOS can be found in the vicinity of the Fermi level for the Ru interstitial coordinated by three oxygen atoms. Local atomic environments for the investigated configurations are shown at the top of the respective panels.

Now, we examine the electronic structure of pure and defect containing SrRuO_3_. In Fig. 5(a–e), we have plotted the atom projected density of states (DOS) of the bulk and various interstitial and anti-site pair defects to investigate the impact of interstitial defects on the electronic structure of orthorhombic SrRuO_3_. Figure 5(a–e) reveals that in the vicinity of the Fermi level, a large portion of the DOS originate mainly from Ru atoms. The analysis of the orbital-projected DOS (Fig. 5a) indicates that in both majority and minority spin channels, the Ru ions exhibit 4(*d*) character. Particularly t_2*g*_, bands near the Fermi level (from ~−1.2 to 1.2 eV) (also see Supplementary Fig. 1). While electronic states with e_*g*_ symmetry are found above 1.2 eV and below −3 eV of the Fermi level. In the majority spin channel, all interstitial defects generate a crossover of the t_2*g*_ bands at the Fermi level. In the case of the oxygen interstitial and the antisite pair (Ru-Sr) defects, in the minority spin channel, the t_2*g*_ orbitals are found right below the Fermi level. While in the case of Ru and Sr interstitial, the t_2*g*_ orbitals are found above the Fermi level. In case of oxygen interstitial, a significant *p*-*d* hybridization between the relatively extended Ru (4*d*) and O (2*p*) orbitals is observed (see Fig. 5b). This is an intriguing finding, as identifying a method of selectively controlling these defects would open a new realm of modifying electronic structure with never before possible post-growth precision.

## Discussion

Given the fact that defects are inevitable, even in high-quality films, there is always a finite concentration of structural and compositional defects. The impact of defects on functionality in complex materials is largely unknown due to our inability to quantify and selectively manipulate them in a controlled fashion. Overcoming this hurdle is one of the outstanding challenges of condensed matter physics. The complex nature of oxides allows various kinds of defects for a range of concentrations. Defects are expected to form in collisions during implantation. Sometimes, conventional techniques (i.e. X-ray diffraction) fail to detect small concentration of defects in films^6^. However, in recent experimental observations, it has been demonstrated that even an undetectable concentration of defects can potentially tune the functionalities of perovskite thin films^6^. The possible existence of interstitials (He, O and Ru) and vacancies defects (oxygen and Ru) in constrained orthorhombic SrRuO_3_ is strongly suggested by our calculated formation energies (see Table 1), which are much smaller than the ones of the implanted He. It is also evident from the lower formation energies that the generation of intrinsic defects is favored in the presence of oxygen vacancies. Our computational study demonstrates that sputtering techniques such as ion bombardment or implantation can certainly lead to the formation of isolated point defects, clusters, and complexes. The question is “how many and to what extent can these different defects be controlled?” The ability to control specific defects in these materials would be extremely valuable to fundamental studies aimed at disentangling defect contributions to functionality and allow new levels of control over correlated behaviors.

The intrinsic defects (O_*vac*_, O, Ru interstitial) which break the RO_6_ octahedral symmetry disrupt the structural and electronic properties of SrRuO_3_ resulting in large changes in the materials properties (see Fig. 5). The present theoretical work reproduces the expansion of out-of-plane lattice parameters observed in recent experimental measurements while demonstrating that the observed lattice expansion is likely the result of secondary defect formation outside of the previously suggested single operator He interstitial mechanism^5, 6^. A series of calculations were performed with varying concentration of intrinsic defects and He interstitials (Fig. 1b). According to theory, the recognizable expansion of the lattice may result primarily from the local chemical environment and distortion in the lattice introduced by the oxygen interstitials.(see Fig. 1(b)). Our work shows that O interstitials are more likely to form than cation interstitials because of their lower formation energy. Hundreds of these defects could be formed with a single implanted He atom introduced at energies >4 keV.

Defects affect the chemical bonding as well as the local structural environment. Therefore, they can be used to tailor the properties of pristine materials. The presence of defects can be unveiled by using many experimental techniques^3^. As shown in the top panel of Fig. 5, the presence of defects significantly affects the RuO_6_ octahedra along the c axis. The distortion of the oxygen cages introduced due to the formation structural defects (isolated point defects, clusters and complexes) opens the gap inside t_2*g*_ and e_*g*_ bands, forcing the t_2*g*_ electrons of Ru to split, this splitting can be measured experimentally. In the case of SrRuO_3_, the distortion of the oxygen octahedral is absent, and no gap is observed in Fig. 5a. In all cases, a close examination of the orbital-projected DOS clearly reveals that Ru (4*d*) bands determine the electronic structure near the Fermi level. In addition, measurements on specific heat^35^, optical properties^36^, infrared and optical reflectivity^37^, thermal, magnetic, and transport properties^38^, and photo-emission and x-ray absorption spectroscopy^39–43^ have confirmed the impact of Ru 4*d* orbitals on the properties of Ru-based oxides. Thus, our study shows that these orbitals can be largely modified by defects induced by the ion implantation process.

Sputtering techniques such as ion bombardment or implantation can potentially lead to the formation of isolated point defects, clusters, and complexes^44^. The effects of He implantation on electronic and structural properties can be a complicated process, which includes the interaction of structural defects with the implanted He atoms and of the implantation-induced strain. By using irradiation of ion beam, the deposited He atoms and irradiation-induced structural damage are distributed according to the depth near the surface. But in the practical situation, the distributions of He atoms and structural damage are nearly uniform in a section of the material^31–34^. Moreover, for the irradiation of ion beam, both the He atoms and structural damage are introduced in a quite short time compared to the annealing time. Therefore, the defects and their properties are likely to have some differences between the two circumstances. Therefore, understanding the behavior of He and the evolution of structural damage after irradiation of ion beam requires further investigation.

There are three predominant inherent point defect types present in all crystalline materials: vacancies, interstitials, and site reversals or antisite, e.g. some lattice sites contain vacancies, some elements are displaced into interstitial positions and, in multi-component materials, constituent atoms may sit in the “incorrect” positions, eg. A-site/B-site reversal in an ABO_3_ perovskite. These defects may be relatively low in high purity samples but are always present^45^. Helium implantation may induce the exact same types of defects through knock-on damage. Here, an incident ion’s nucleus collides with a resident nucleus in the target crystal which passes energy to the lattice. This can unseat the resident atom creating a vacancy while driving the now free atom to an interstitial position or causing it to land in an open vacancy position, which was previously vacated by an atom of another species. Thus, all of the 3 predominant defect types present in “perfect” as-grown TMO crystals might also be induced by ion implantation. We propose future experimental efforts in which as-grown films are gradient dosed across the film surface. This would offer an exciting new avenue of addressing disorder effects in materials, as it would offer never before possible access to general trends in electronic structure and defect concentrations as a function of dose in the same film. This would allow for a desperately needed built-in control region which would greatly aid in the modeling of functional modification under changing defect concentrations.

## Conclusions

In conclusion, we investigated the effects of He implantation on the electronic and structural properties of SrRuO_3_. We show that it is a complicated process which includes generation and interaction of point defects, by the implanted He atoms which result in implantation-induced strain. A systematic step-by-step computational exploration of commonly considered point defects (i.e., interstitials, including self-interstitials, substitutional atoms, and vacancies) has lead us to new insights on the unexplored territory of the possible intrinsic defects resulting from He implantation on electronic and structural properties of perovskite thin films. The present work is inspired by recent He implantation experiments, which demonstrated that the application of low doses of low energy He ions can tune the functionality of perovskite thin films. We find that though He interstitials could produce the structural changes observed experimentally, the dose required is several orders of magnitude larger than the one reported experimentally. In contrast, several intrinsic defects, with a formation energy below 5 eV, could be created by collisions with a single implanted He atom (at energies of ~4–10 keV). At low doses O, Sr, and, Ru interstitials produce much larger expansion in the lattice constants than He. Vacancies produce effects comparable to He. These defects can generate lattice expansion, octahedral rotation, hole conduction, ferroelectric performance, lowering the carrier mobility, etc. We find that migration barriers for the different induced defects vary and are thus susceptible to the use of post-implantation annealing to modify specific defect ratios. This finding opens a new realm of possibility in helping to disentangle specific defect contributions and to designing functionality through controlled defect evolution.

## Methods

### Computational Details

The quantum mechanical computations were performed using density functional theory (DFT)^46, 47^ as implemented in the Vienna *ab initio* software package^48, 49^. The generalized gradient approximation (GGA) functional parametrized by Perdew, Burke and Ernzerhof (PBE)^17^ to treat the electronic exchange-correlation interaction, the projector augmented wave (PAW)^50^ potentials, and plane-wave basis functions up to a kinetic energy cutoff of 500 eV were employed. Although, the density of states (DOS) for constrained undoped SrRuO_3_ are in close agreement with prior results performed at a same level of theory^18, 19, 51^. The minimum (DFT) energy pathways are calculated using the generalized solid-state nudged elastic band method^25^. In present work, we did not considered the Van der Walls interactions. Moreover, we expect these corrections to be smaller than 0.05 eV for defect formation energies.

Given that the semilocal electronic exchange- correlation functional adopted here places an uncertainty in the energetic position of dopant-derived defect levels, we make the caveat that the following conclusions should be viewed as qualitative. While it may be desirable to perform more sophisticated computations (e.g., using hybrid functionals) to ascertain the validity of the emerging notions pertaining to the electronic structure, these have not been attempted in the present work. At the atomic scale, the local chemical bonding and structure of a defect containing system can be unveiled using many analytical techniques.

In order to study the effects of He implantation on the structural properties of epitaxial films, we perform calculations on a of SrRuO_3_ model system. Our systematic first-principles calculations starts with the *Pbnm* ground state of SrRuO_3_, containing 4 formula units or 20 atoms per cell. We fixed the *a* and *b* lattice vectors to match a SrTiO_3_ substrate considered in many recent experimental studies^5, 7^. It is worth mentioning here that the constrained-bulk framework is subsequently used in first-principles calculations for perovskite oxide thin films and superlattices^18, 19, 52^.

## Electronic supplementary material

SUPPLEMENTARY INFO
