# Supplementary material for: Designing functionality in perovskite thin films using ion implantation techniques: Assessment and insights from first-principles calculations
Source: Sci Rep. 2017 Sep 11;7:11166. doi: 10.1038/s41598-017-11158-4 (PMC5593984; doi:10.1038/s41598-017-11158-4)
Supplement: Supplementary file 1 — SUPPLEMENTARY INFO [file 41598_2017_11158_MOESM1_ESM.pdf]

Designing functionality in perovskite thin films using ion implantation techniques: Assessment and insights from first-principles calculations

Vinit Sharma,[1] Andreas Herklotz,[1] Thomas Zac Ward,[1] and Fernando A. Reboredo[1*]

[1] *Materials Science and Technology Division, Oak Ridge National Laboratory, Oak Ridge 37831-6056, TN, USA*

Formation energies

The defect chemistry and the effects of He implantation on structural and electronic properties of $SrRuO_3$ are calculated using the projector augmented plane wave basis functions with polarizations as implanted in the Vienna *ab initio* simulation package (VASP).[1–3]
To evaluate the energetics of defect formation, we define the 0 K defect formation energy, E_f^d, as

$$E_f^d = E_{SRO}^d - E_{SRO} + \mu^d - \mu^{He} \tag{1}$$

where E_{SRO}^d and E_{SRO} are, respectively, the DFT energies of the with and without defect SRO supercells, and μ^{He} and μ^d are the elemental chemical potentials of the He and the host (Sr/Ru/O) atoms. In addition, a separate set of calculations involving an O_{vac} adjacent to the defect was also performed, in order to probe the tendency for defect-O_{vac} cluster formation. The combined formation energy of a defect *and* an O_{vac} ($E_f^{d-O_{vac}}$) was determined as

$$E_f^{d-O_{vac}} = E_{SRO}^{d-O_{vac}} - E_{SRO} + (\mu^d - \mu^{He} - \frac{1}{2}\mu_{O_2}) \tag{2}$$

where $E_{SRO}^{d-O_{vac}}$ is the DFT total energy of the supercell containing a defect and an adjacent O_{vac}, and the oxygen chemical potential (μ_{O_2}), is taken to be the DFT energy of an isolated O_2 molecule in the gas phase. We also note that the O_{vac} formation energy, $E_f^{O_{vac}}$, in any given case of defect (and site) is simply given by the difference of the above two formation energies, *i.e.*,

$$E_f^{O_{vac}} = E_f^{d-O_{vac}} - E_f^d \tag{3}$$

The formation energies defined above depend on the particular choice of the atomic chemical potentials. In the present study, the chemical potential of the Ru and Sr atoms, ($\mu_{Ru/Sr}$), is defined using the total energies of their most stable oxides namely RuO_2 or SrO for oxygen rich conditions. as

$$\mu^M = \frac{1}{y}\left(E_{M_yO_x} - \frac{x}{2}\mu_{O_2}\right) \tag{4}$$

where $E_{M_yO_x}$ is the DFT energy of the most stable oxide M_yO_x, where M represents either Ru or the Sr atom, and x and y represent the stoichiometry of the oxide. Furthermore, the formation energy of the defects and host simple oxide may be defined as

$$\begin{aligned} E_f^{M_yO_x} &= \frac{1}{y}\left(E_{M_yO_x} - \frac{x}{2}\mu_{O_2} - yE_{Bulk}\right) \\ &= \mu^M - E_{Bulk} \end{aligned} \tag{5}$$

where E_{Bulk} is the DFT energy of the most stable bulk elemental solid formed either from Ru or Sr atom.

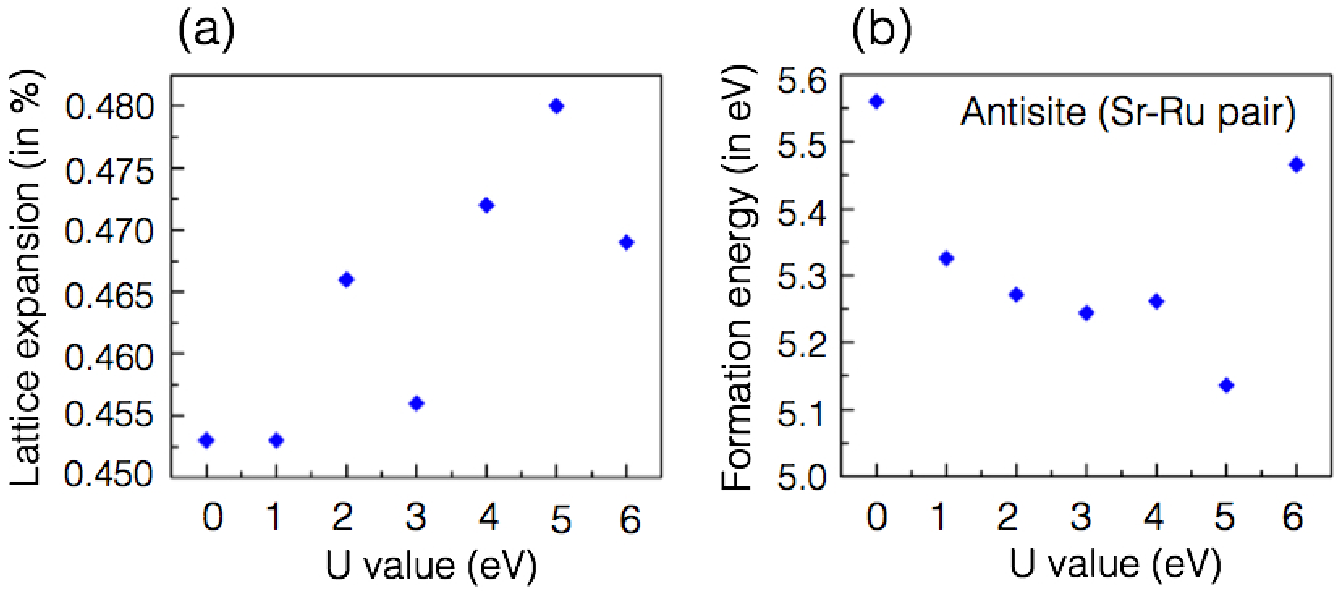

Figure 1: (Color online) Dependence of the **(a)** lattice expansion and **(b)** formation energies, for antisite (Sr-Ru pair) defect on the Hubbard parameter U.

It is a well known deficiency that GGA introduces a nonphysical electron self-interaction energy.[8] The accuracy of the GGA computed formation energies is attributable to the cancellation of this self-interaction energy between different calculations. Prior to investigating defects in SRO, we first desire to confirm the level of accuracy that may be expected at the level of theory used here (namely, the semilocal PBE exchange-correlation functional). One method to mitigate the self-interaction error is the GGA + U method, which sometimes correctly predicts the relative energetics, magnetic ground states, and electronic structure for systems in which GGA fails.[9,10]

Here, we compare the formation energies for antisite (Sr-Ru pair) defect and resultant lattice expansion as a function of U value. Such a comparison, portrayed in Supplementary Fig. 1, immediately shows that for a range of U value (U= 0 to 6), our GGA-computed results differ by 0.4 eV for formation energies, and by 0.03 $\AA$ for lattice expansion. Therefore, in present work we used the semilocal PBE exchange-correlation functional.

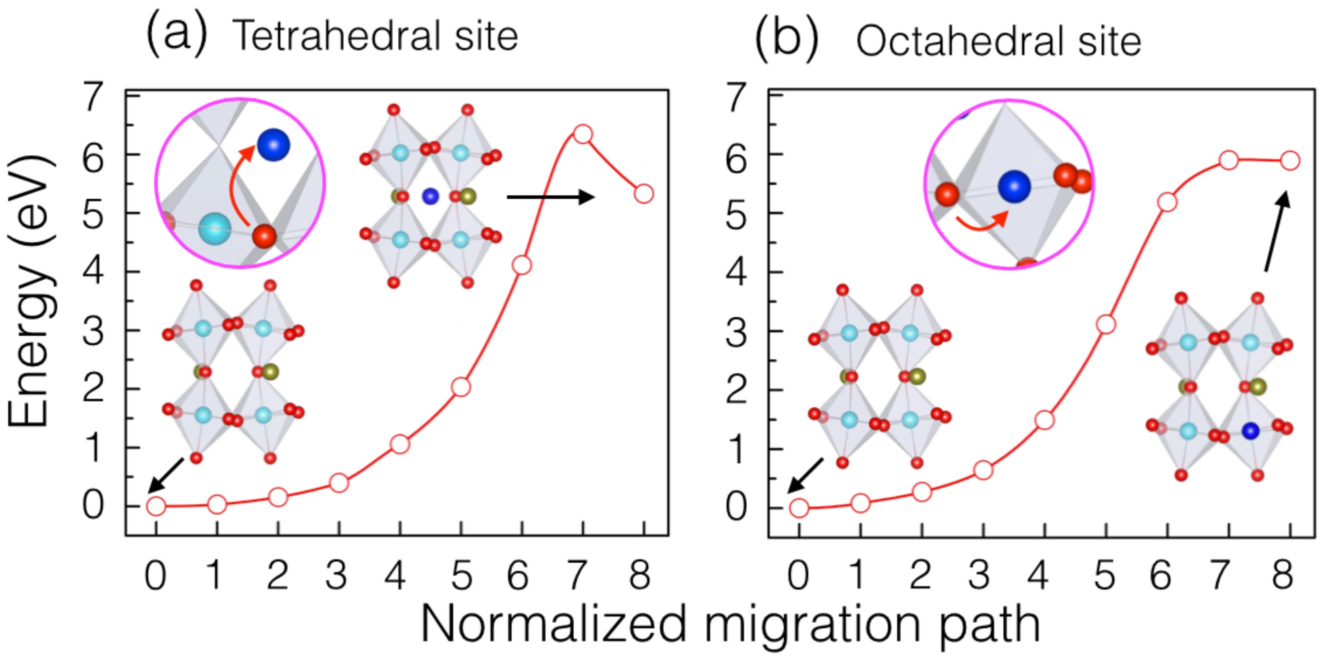

Figure 2: (Color online) **Computed energy barriers for oxygen interstitial migration (a)** O interstitial migration to tetrahedral site. **(b)** O migration to octanedral site. He, O, Sr, and Ru atoms are shown in blue, red, dark yellow and cyan, respectively. In the inset, schematic illustration of the initial and final configurations used in the barrier calculations is shown. The lines are guides for the eye only.

---

* Electronic address: `sharmavk1@ornl.gov,bfr@ornl.gov`

[1] G. Kresse and J. Hafner, Phys. Rev. B **49**, 14251 (1994).

[2] G. Kresse and J. Furthmuller, J. Comput. Mater. Sci. **6**, 15 (1996).

[3] G. Kresse and J. Furthmuller, Phys. Rev. B **54**, 11169 (1996).

[4] J. P. Perdew, K. Burke, and M. Ernzerhof, Phys. Rev. Lett. **77**, 3865 (1996).

[5] S. Curtarolo, D. Morgan, and G. Ceder, Calphad **29**, 163 (2005).

[6] H. J. Monkhorst and J. D. Pack, Phys. Rev. B **13**, 5188 (1976).

[7] P. E. Blöchl, O. Jepsen, and O. K. Andersen, Phys. Rev. B **49**, 16223 (1994).

[8] V. I. Anisimov, J. Zaanen, and O. K. Andersen, Phys. Rev. B 44, 943 (1991).

[9] V. I. Anisimov, F. Aryasetiawan, and A. I. Lichtenstein, J. Phys. Condens. Matter 9, 767 (1997).

[10] Anubhav Jain, Geoffroy Hautier, Shyue Ping Ong, Charles J. Moore, Christopher C. Fischer, Kristin A. Persson, and Gerbrand Ceder, Phys. Rev. B 84, 045115 (2011)
